# Real-World Treatment Utilization and Patterns of Adjuvant Olaparib for Early-Stage Breast Cancer: Insights from the Komodo Research Database

**DOI:** 10.3390/curroncol33100589

**Published:** 2026-09-30

**Authors:** Felipe Batalini, Qixin Li, Jennifer F. Hayes, Kathryn Mishkin, Neel Vaidya, Dana Stafkey, Eileen Farrelly, Xiaoqing Xu

**Affiliations:** 1 Mayo Clinic Hospital, Phoenix, AZ 85054, USA; 2 AstraZeneca, Gaithersburg, MD 20878, USA; sandy.li1@astrazeneca.com (Q.L.); jennifer.hayes1@astrazeneca.com (J.F.H.); xiaoqing.xu1@astrazeneca.com (X.X.); 3 Merck Sharp & Dohme LLC, A Subsidiary of Merck & Co., Inc., Rahway, NJ 07065, USA; kathryn.mishkin@merck.com; 4 Cencora, Conshohocken, PA 19428, USA; neel.vaidya@cencora.com (N.V.); dana.stafkey@cencora.com (D.S.); eileen.farrelly@cencora.com (E.F.)

**Keywords:** early breast cancer, olaparib, gBRCAm, BRCA mutation, claims analysis, adherence, discontinuation, combination patterns, triple-negative breast cancer, hormone-receptor-positive

## Abstract

**Background and Objectives:** As adjuvant treatment for human epidermal growth factor receptor 2 (HER2)-negative early-stage breast cancer (eBC) evolves, real-world data should inform olaparib utilization, sequencing, and combination patterns in clinical practice. **Methods:** Using Komodo US claims data (1 January 2019–31 December 2023), we retrospectively analyzed HER2-negative eBC patients on adjuvant olaparib. Continuous insurance for 12 months pre- and ≥30 days post-index date (first olaparib prescription), with follow-up through disenrollment or study end, was required. Outcomes, including time on therapy (Kaplan–Meier median persistence), adherence (proportion of days covered), dose reduction, and combination patterns, were stratified by claims-derived hormone-receptor-positive (HR+) and triple-negative BC (TNBC) status. **Results:** Among 176 olaparib-treated patients (median age, 44 years), the median (95% CI) time on therapy was 11.0 (9.2, 12.2) months; 27.3% (n = 48) discontinued before 12 months without re-initiating, 19.9% experienced a dose reduction, and median adherence was 90%. Olaparib was combined with other therapies in 48.3% (n = 85) (29.6% [32/108] of TNBC and 77.9% [53/68] of HR+ patients), most often with immunotherapy in TNBC (81.3%, n = 26) and endocrine therapy in HR+ (88.7%, n = 47). **Conclusions:** Real-world data suggest favorable persistence and dosing patterns with adjuvant olaparib. Future research should prioritize olaparib combination regimen safety and effectiveness.

## Simple Summary

Breast cancer is the most common cancer among women, and those carrying inherited mutations in their *BRCA1* or *BRCA2* genes face a significantly higher lifetime risk of developing the disease. Olaparib, a targeted therapy that blocks proteins that cancer cells rely on for DNA repair, was approved in 2022 for use after surgery in high-risk, HER2-negative early-stage breast cancer patients with these inherited mutations. While clinical trials have demonstrated substantial survival benefits, limited real-world data exist on how olaparib is used in routine clinical practice. This study analyzed US insurance claims data and found that, among 176 olaparib-treated patients, most remained on treatment for the recommended 12-month course, while few required dose reductions or permanently discontinued therapy. Nearly half of these patients received olaparib alongside other therapies. These findings can help guide clinical decision-making, highlight opportunities to improve patient support, and inform future studies evaluating the safety and effectiveness of olaparib combination regimens.

## 1. Introduction

Early-stage breast cancer (eBC) accounts for 66% of all BC cases in the United States (US) [1]. While the average lifetime BC risk in the US is 13% [2], women with germline mutations in the *BRCA1* and/or *BRCA2* genes (gBRCAm) have an increased risk, ranging from 60% to 70% [3,4,5,6]. Despite an overall 5-year survival rate of 92.4% to 100% for patients with human epidermal growth factor receptor 2 (HER2)-negative localized BC [7], high-risk patients face a substantially higher recurrence risk. Olaparib, a poly (adenosine diphosphate-ribose) polymerase (PARP) inhibitor, was approved as an adjuvant treatment for HER2-negative eBC with gBRCAm, but real-world data on adjuvant treatment utilization are limited.

While real-world data have revealed poor adherence to adjuvant hormonal treatment [8,9,10], data on more recent treatments, such as cyclin-dependent kinase 4/6 (CDK4/6) inhibitors (e.g., abemaciclib), immunotherapy (pembrolizumab), and chemotherapy (capecitabine), are limited [11,12,13,14]. As new treatments emerge, analyzing their utilization is crucial for assessing tolerability and adherence, since clinical impact depends on these factors. Specifically, PARP inhibitors have gained importance in eBC due to high recurrence and mortality rates among gBRCAm patients [15,16]. In the OlympiA trial (ClinicalTrials.gov NCT02032823), which led to the FDA approval of olaparib (Lynparza^®^) on March 11, 2022, for high-risk gBRCAm HER2-negative patients who had previously received chemotherapy [17], adjuvant olaparib significantly increased invasive-disease-free survival (hazard ratio, 0.58; 99.5% CI, 0.41–0.82; *p* < 0.001) and reduced the risk of death by 32% (hazard ratio, 0.68; 99% CI, 0.47–0.97; *p* = 0.009) compared to a placebo [18]. In an exploratory analysis with a median follow-up of 6.1 years, olaparib showed a sustained 28% reduction in death risk compared to a placebo (hazard ratio, 0.72; 95% CI, 0.56–0.93) [19]. While the trial showed an overall survival benefit, using real-world data to understand clinical use is important to identify utilization patterns, which can be used to optimize treatment strategies.

The fact that therapies change during the conduct of trials creates a clinical problem. It is often challenging to reconcile combination and sequencing strategies, let alone estimate the clinical benefit of these different strategies. Since olaparib was not approved during the CREATE-X (capecitabine), KEYNOTE-522 (pembrolizumab), and monarchE (abemaciclib) trials [20,21,22], there are no published data on its combination or sequencing with these therapies in practice [23]. The National Comprehensive Cancer Network (NCCN) guidelines note that sequencing or combining adjuvant pembrolizumab with olaparib may be considered in early triple-negative BC (TNBC). Further, the benefit and optimal sequence of adjuvant olaparib and CDK4/6 inhibitors, such as abemaciclib, in high-risk HR+ disease is unknown [24]. However, international guidelines recommend sequencing olaparib ahead of CDK4/6 inhibitors for patients with gBRCAm [25,26].

Real-world data supporting combinatory or sequential use with olaparib may facilitate clinical decision-making. Additionally, real-world data in sub-populations, like patients with TNBC (nearly 11% of cases) [7], may differ from the overall cohort. Moreover, racial disparities are associated with differences in time to diagnosis and treatment, access to care, survival and BC subtypes [27,28,29,30]. Therefore, real-world data that allow stratified analyses of olaparib utilization in these subgroups can help improve patient outcomes and tailor treatment strategies.

Herein, we aim to leverage data from the Komodo Research Database to describe clinical utilization and adherence, dosing, and combination patterns of adjuvant olaparib use.

## 2. Materials and Methods

### 2.1. Study Design

An observational cohort study was designed using HER2-negative eBC patients identified from Komodo Research Database. The study period was from 1 January 2019 to 31 December 2023. Patients were followed from olaparib initiation until disenrollment (i.e., loss or gap in insurance coverage) or end of the study period, whichever event occurred first.

### 2.2. Data Source

Data from the Komodo Research Database included de-identified data on more than 320 million patients enrolled in US health plans since 2012, more than 140 million of whom had closed claims from more than 150 payers and over 3.5 million providers. The database is fully de-identified and compliant with the Health Insurance Portability & Accountability Act (HIPAA). Closed claims are encounters sourced directly from payers to provide a complete patient journey, including full medical and/or prescription benefit information.

Diagnoses and procedures for outpatient and inpatient visits/stays were identified by International Classification of Diseases, Tenth Revision, Clinical Modification (ICD-10-CM) diagnostic codes; International Classification of Diseases, Tenth Revision, Procedure Coding System (ICD-10-PCS); Current Procedural Terminology (CPT); and Healthcare Common Procedure Coding System codes. Outpatient pharmacy claims were captured by National Drug Codes (NDCs). Race and ethnicity data collected from the Komodo Research Database were sourced from clinical electronic health records, patient intake forms, and payer enrollment files.

### 2.3. Study Population

We included adults (≥18 years) with ≥1 inpatient or ≥2 outpatient claims, ≥30 days but ≤365 days apart, containing a BC diagnosis (ICD-10 code C50.xx) on non-diagnostic medical claims during the study period. The first diagnosis date was defined as the initial BC diagnosis, and patients needed evidence of BC surgery (via CPT or ICD-10-PCS codes) within 12 months of this date. The index date was the first olaparib prescription claim (identified via NDC) on or after 11 March 2022 (FDA approval), occurring after the first BC diagnosis and surgery date. Patients needed ≥12 months of pre-index and ≥1 month of continuous medical and pharmacy insurance enrollment post-index. Those with metastatic disease (ICD-10 codes: C77.xx–C79.xx; C80.0; C7B.xx) or those receiving HER2-targeted drugs (trastuzumab, ado-trastuzumab emtansine, fam-trastuzumab deruxtecan, pertuzumab, trastuzumab-pertuzumab combination, or margetuximab) or kinase inhibitors (lapatinib, neratinib, or tucatinib), identified via NDC, since data availability (1 January 2019) through the index date, were excluded to retain only non-metastatic patients and patients classified as HER2-negative using a claims-derived proxy. Other exclusions included patients with co-indicated primary cancers (including ovarian [C56.x], prostate [C61.x], pancreatic [C25.x], fallopian tube [C57.0x], and peritoneal cancers [C48.x])—in any position on any claim—during the pre-index period, and clinical trial participants (ICD-10 diagnosis code: Z00.6) seen anytime during the study period. Note: As genetic testing results are not captured in administrative claims data, gBRCAm status could not be directly confirmed. Olaparib utilization in a non-metastatic, HER2-negative breast cancer context was used as an implicit proxy for gBRCAm status, given that olaparib’s FDA-approved adjuvant indication is restricted to gBRCAm, HER2-negative, high-risk early breast cancer—a designation requiring confirmed mutation status prior to prescribing in clinical practice.

The following sub-cohorts were evaluated: (a) neoadjuvant treatment (evidence of chemotherapy, targeted therapy, immunotherapy, hormonal or radiation therapy before BC surgery date); (b) platinum-based chemotherapy (evidence of carboplatin or cisplatin-based chemotherapy regimen in the neoadjuvant or adjuvant setting, prior to index date); (c) race and ethnicity (White non-Hispanic; Hispanic or Latino; African American; and Hispanic or Latino and African American, plus all other races and ethnicities combined [sum of Asian and Pacific Islander and Other]); (d) HR/TNBC (HR+: for the purposes of this claims analysis, ≥1 prescription claim for hormonal therapy during the study period was used for classification as “claims-derived HR+”; TNBC: for the purposes of this claims analysis, no prescription claims for hormonal therapy during the study period was used for classification as “claims-derived TNBC”).

### 2.4. Study Outcomes

Study outcomes evaluated during the follow-up period included utilization patterns: (a) Interruption, defined as a gap in prescription fills of 14 to 55 days without olaparib, the first date of which was the interruption date. (b) Discontinuation, defined as a gap in fills ≥56 days (i.e., 2 consecutive missed fills) [31] without olaparib. The first day of a gap was defined as the discontinuation date, and persistence was the time from the index date to discontinuation. Notably, we assessed Kaplan–Meier (KM)-calculated median persistence as a surrogate for time on olaparib therapy. (c) Composite of interruption and discontinuation, defined as the first occurrence of either. (d) Re-initiation, defined as evidence of restarting olaparib after discontinuation. (e) Dose reduction, defined as a decrease in dose from the previous pharmacy claim. (f) Treatment adherence outcomes included the proportion of days covered (PDC), calculated as the number of unique days of supply divided by the total days from the index date to the last day of olaparib use in the follow-up period, and PDC ≥80% was deemed “adherent”. (g) Adjuvant olaparib treatment pattern outcomes included (i) combination therapy, defined as any treatment (targeted, immunotherapy, chemotherapy, hormonal, and/or radiation) overlapping ≥1 day with olaparib, and (ii) monotherapy, defined as olaparib use without overlapping treatments during the days’ supply period.

### 2.5. Statistical Analysis

Descriptive analysis was conducted for all variables, including demographic, clinical, and outcome variables. Descriptive statistics were tabulated in the form of frequency counts and percentages for categorical variables, and mean, standard deviation, median, and 25th and 75th percentiles for continuous variables. KM analysis was conducted for all time-to-event utilization outcomes. Median and 95% confidence intervals (CIs) were presented for KM-calculated time-to-event outcomes. Sankey diagrams were constructed to illustrate adjuvant therapy before, during, and after olaparib use. This study used Statistical Analysis Software (SAS; Enterprise Guide version 9.4, SAS Institute; 2023; Cary, NC, USA) for analysis.

## 3. Results

Overall, we identified 4768 adult patients diagnosed with BC who were prescribed olaparib and classified as HER2-negative in the Komodo Research Database. To select an eBC cohort, we retained non-metastatic patients (based on available data through the index date), resulting in a sample of 990 patients. After satisfying other inclusion and exclusion criteria, we identified 176 eBC patients treated with olaparib, who had a median follow-up of 9.5 months and comprised the final sample for our analysis.

### 3.1. Baseline Demographic and Clinical Characteristics

The median age of the population was 44 years (range, 24 to 81). Patients with claims-derived TNBC (n = 108) were older than those with claims-derived HR+ disease (n = 68) (median, 48 years [range, 24 to 81] vs. 40 years [range, 28 to 75], *p* = 0.001). These patterns were similar to those seen in a US Surveillance, Epidemiology and End Results (SEER) Program database analysis [32]. The demographic breakdown of our population was as follows: 39.8% were White non-Hispanic, 11.9% were Hispanic or Latino, 9.1% were African American, and a minority (2.3%) were Asian and Pacific Islander.

The most frequent comorbidities included hypertension (37.5%), depression (28.4%), and dyslipidemia (23.3%). Overall, 74.4% had a mastectomy as the only surgery. Neoadjuvant therapy was used in 69.9% of cases; 31.3% of patients received chemoimmunotherapy, 27.3% received chemotherapy alone, 5.1% received chemotherapy plus hormonal therapy, and 4.6% received chemoimmunotherapy plus hormonal therapy. Baseline demographic and clinical characteristics appear in Table 1, while Table S1 shows clinical characteristics stratified by the presence of neoadjuvant treatment and platinum-based chemotherapy.

### 3.2. Time on Therapy

Overall, the median time on therapy (95% CI) (i.e., KM-calculated time to first treatment discontinuation) among all patients was 11.0 (9.2, 12.2) months. Among claims-derived HR+ (n = 68) and claims-derived TNBC patients (n = 108), the median time on therapy was 12.0 (9.2, 12.9) months and 10.0 (6.0, 12.0) months, respectively (Table 2). Among neoadjuvant or adjuvant platinum (n = 80) and non-platinum users (n = 96), the median time on therapy was 9.2 (4.9, 12.5) months and 11.2 (9.4, 12.5) months, respectively (Table S2).

### 3.3. Adherence, Dosing and Discontinuation

**Figure 1:**
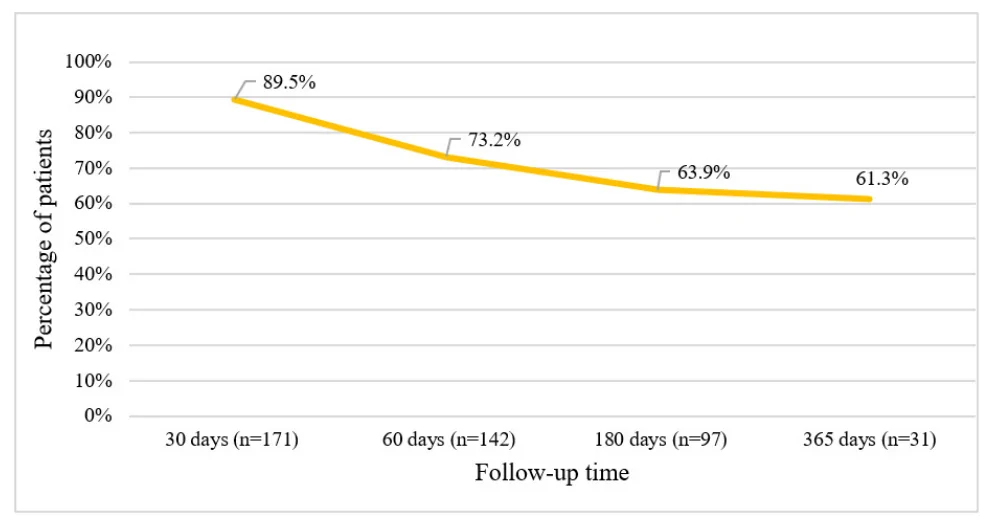
Adherence to adjuvant olaparib at different follow-up timepoints. Key: PDC—proportion of days covered. Note: PDC was calculated at each timepoint among the population who were still-enrolled olaparib users and did not drop out of the study, which is why sample sizes at later timepoints were lower.

Overall, the median (25th percentile [P25], 75th percentile [P75]) adherence of all patients with ≥30 days of follow-up was 90% (80%, 100%). Patients stratified by neoadjuvant treatment, use of platinum therapy or claims-derived HR+/TNBC status showed similar findings (Table 3). Overall, adherence ranged from 89.5% to 63.9% at months 1 to 6, respectively. Figure 1 presents overall adherence trends over treatment time. Olaparib adherence dropped between months 1 and 2 of follow-up but remained stable between months 6 and 12. No notable differences were observed by race and ethnicity or prior platinum therapy subgroups.

Out of 166 patients with non-missing pharmacy data, 19.9% (n = 33) had a dose reduction, with a median time to first dose reduction of 2.6 months. Overall, 27.3% discontinued treatment before the recommended duration and did not re-initiate therapy; 42.0% (n = 74) discontinued treatment within 12 months of therapy initiation, 30.6% (n = 26) of whom subsequently re-initiated therapy. When stratified by race and ethnicity, 45.7% (n = 32) of White non-Hispanic patients and 41.8% (n = 23) of the group composed of Hispanic or Latino and African American patients, plus all other races and ethnicities combined (i.e., Asian and Pacific Islander and Other), discontinued olaparib before 12 months, while 16.7% (n = 6) of White non-Hispanic patients and 46.4% (n = 13) of the group composed of Hispanic or Latino and African American patients, plus all other races and ethnicities combined, re-initiated again. Among those who re-initiated olaparib overall, the median (P25, P75) time to re-initiation was 2.5 (2.0, 3.1) months. Table 2 shows all utilization outcomes.

### 3.4. Monotherapy vs. Combination Use

**Figure 2:**
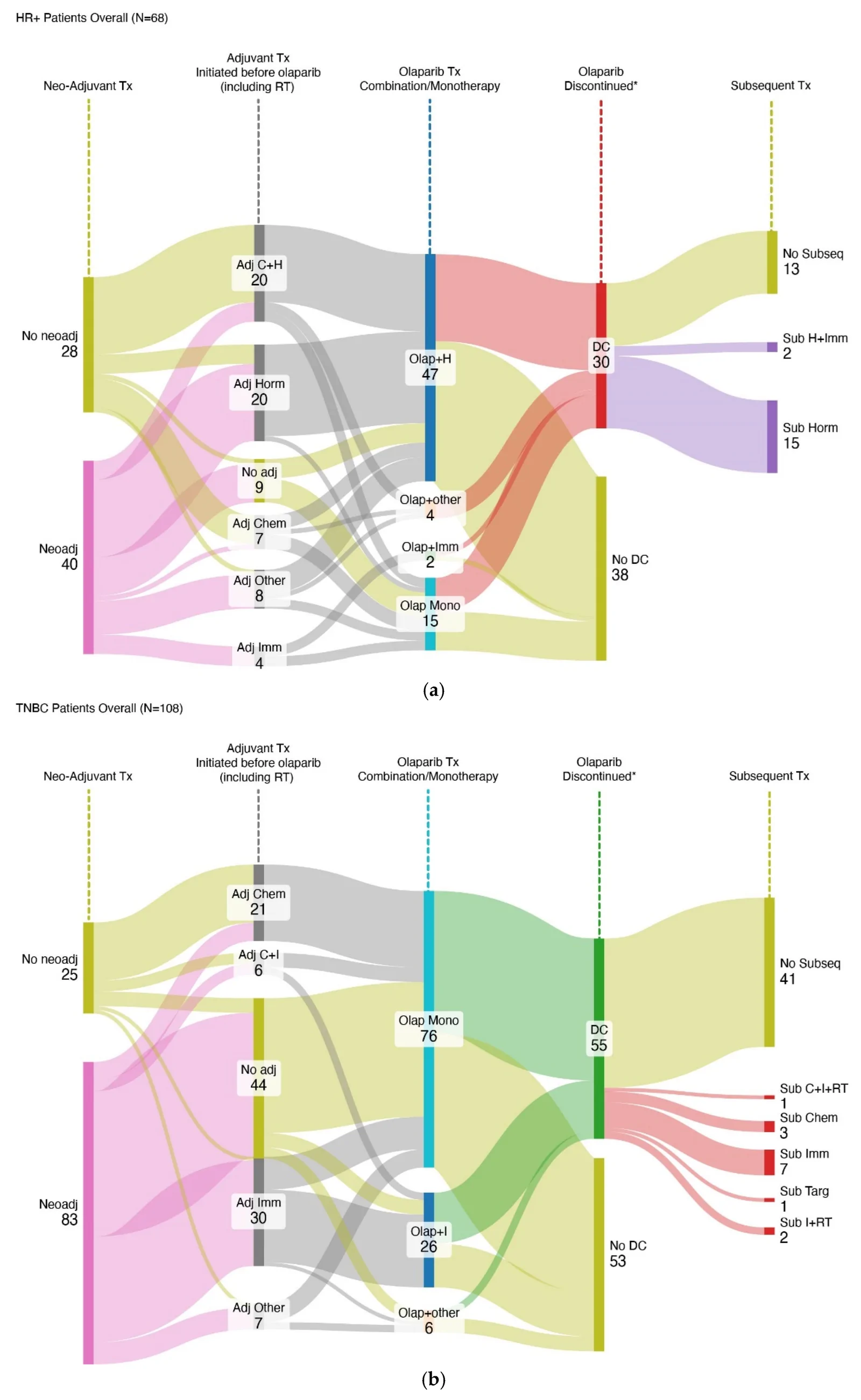
(**a**) Sankey diagram of adjuvant treatments before and during olaparib treatment among claims-derived HR+ patients (N = 68), and (**b**) Sankey diagram of adjuvant treatments before and during olaparib treatment among claims-derived TNBC patients (N = 108). Key: Adj—adjuvant treatment; Adj Chem—adjuvant chemotherapy; Adj Horm—adjuvant hormonal; Adj Imm—adjuvant immunotherapy; Adj Other—adjuvant other treatment; Chem—chemotherapy; DC—discontinued treatment; HR+—hormone-receptor-positive; TNBC—triple-negative breast cancer; Imm—immunotherapy; Neoadj—neoadjuvant treatment; Olap Mono—olaparib monotherapy; Olap + H—olaparib plus hormonal treatment; Olap + Imm—olaparib plus immunotherapy; RT—radiation therapy; Sub H + Imm—subsequent hormonal plus immunotherapy; Sub Horm—subsequent hormonal therapy; Sub C + Imm + RT—subsequent chemotherapy plus immunotherapy plus radiation therapy; Sub Chem—subsequent chemotherapy; Sub Imm—subsequent immunotherapy; Sub Imm + RT—subsequent immunotherapy plus radiation therapy; Sub Targ—subsequent targeted therapy; Subseq—subsequent treatment among those that discontinued after last olaparib claim seen; Tx—treatment. * Discontinued patients represent discontinuation during the entire study period. Note: Adj Other for HR+ (n = 8) includes adjuvant capecitabine plus hormone therapy plus immunotherapy (n = 2), adjuvant chemotherapy (other than capecitabine) plus hormonal therapy plus immunotherapy (n = 2), adjuvant chemotherapy (other than capecitabine) plus targeted therapy (n = 1), adjuvant hormonal plus immunotherapy (n = 2), and adjuvant radiation therapy (n = 1). Olap + other (n = 4) includes olaparib combined with chemotherapy plus immunotherapy (n = 1), chemotherapy plus hormonal therapy (n = 2), and hormonal plus immunotherapy (n = 1). Note: Adj Other for TNBC includes radiation therapy (n = 7); Olap + other (n = 6) includes olaparib combined with chemotherapy and immunotherapy (n = 1), and radiation therapy (n = 5).

Overall, 48.3% of patients (n = 85) received olaparib in combination with other treatments. Among patients with claims-derived HR+ BC, 77.9% (n = 53) received combination therapy with olaparib, 88.7% (n = 47) of whom were treated with endocrine therapy. Of the remaining six patients, two were treated with immunotherapy, two were treated with chemotherapy plus hormonal therapy, and one patient each was treated with chemoimmunotherapy and hormonal therapy plus immunotherapy, respectively. Among patients with claims-derived TNBC, 29.6% (n = 32) received olaparib combined with other treatments, 81.3% (n = 26) of whom received immunotherapy. Of the remaining six patients, five received radiation, and one received chemoimmunotherapy with olaparib. Figure 2a,b show Sankey diagrams of olaparib treatment patterns, stratified by claims-derived HR+ and claims-derived TNBC patients.

## 4. Discussion

This is the first real-world study using US closed-claims data characterizing demographics, clinical characteristics, and exploring utilization characteristics (including dose reductions, discontinuation, and treatment adherence), and also combination patterns among adjuvant olaparib patients with eBC.

In this cohort, patient age was similar (median, 44 years) to the OlympiA trial [15]; however, White non-Hispanic patients were slightly older (median, 48 years), and the group composed of Hispanic or Latino and African American patients, plus all other races and ethnicities combined, was slightly younger (median, 41 years) than the overall population. Most patients received a mastectomy (74.4%), which reflects a typical choice of risk-reducing surgery in the context of gBRCAm. We note that 7.4% had a lumpectomy followed by mastectomy, possibly due to receiving germline genetic testing results after the initial lumpectomy or an inability to obtain negative margins with the first surgery; however, this inference cannot be confirmed from the current data due to absence of genetic testing data. Nevertheless, these rates are similar to those seen in the literature [33].

As new adjuvant treatments for overlapping populations become available, real-world data on olaparib use are critical for understanding continuity of care. Real-world populations provide greater variability in health status due to barriers to utilization and adherence, including tolerability, cost, and access. Our analysis indicated a median time on therapy of 11 months (95% CI, 9.2, 12.2), which was close to the recommended 12-month duration for olaparib, supporting favorable persistence with olaparib. We find it reassuring that in our cohort, 27.3% discontinued treatment before the recommended duration (without re-initiating), which is comparable to the OlympiA trial’s 25.9%, indicating good translation from clinical trial to the real-world setting. Our real-world cohort may be more susceptible to higher discontinuation rates due to broad-spectrum adverse events [34], limited patient monitoring, and gaps in insurance coverage. Prior real-world studies showed mean adherence rates of 92.3% for abemaciclib and 84% for capecitabine, with varying follow-up times [35,36]. Our study found a mean adherence of 80% for olaparib among patients with ≥30 days of follow-up, with adherence decreasing over time and considerable loss to follow-up at each timepoint evaluated—a pattern often seen in real-world populations. A median time to discontinuation of 2.6 months suggests that enhanced patient support in this initial phase may improve olaparib continuity for the rest of the year.

Dose reductions in BC treatments have been noted for weight changes, drug-related toxicities, or other clinical consequences [37,38]. While olaparib dose reduction has been studied in ovarian cancer [39], this is the first real-world exploration using claims data to provide insights in an eBC cohort. The observed rate of dose reduction of 20% with olaparib was lower than that reported for abemaciclib and capecitabine, which ranged from 37% to 68% [11,12,13,22].

The approval of olaparib as adjuvant therapy generates the need for guidance on sequencing. While both the NCCN Guidelines and the olaparib label state that olaparib can be given concurrently with hormonal therapy [18,24,40], no trial data support combination use with immunotherapy, CDK4/6 inhibitors, or chemotherapy (capecitabine). Trials in the metastatic setting evaluating the combination of PARP inhibitor with immunotherapy demonstrated an acceptable safety profile [41,42,43,44]. Our analysis showed that almost half of our cohort was treated with combination therapy; specifically, a sizeable subset of claims-derived TNBC patients (30%) were treated with olaparib combined with pembrolizumab during follow-up. Based on results from KEYNOTE-522 (KN-522; ClinicalTrials.gov NCT03036488), pembrolizumab is recommended for Stage II–III TNBC [45]. Based on the designs of the KN-522 and OlympiA trials, the concurrent use of pembrolizumab and olaparib has not been tested, resulting in no trial data to support its use. Other options include adjuvant capecitabine, based on CREATE-X trial results (ClinicalTrials.gov NCT00130533), for some early TNBC patients with residual disease after neoadjuvant chemotherapy [22]. However, among 108 claims-derived TNBC cases in our study, only eight patients received capecitabine: six prior to olaparib, one in combination with olaparib, and one after olaparib. This indicates that most patients received olaparib in lieu of capecitabine. Accordingly, efficacy data for capecitabine in patients with early TNBC and gBRCAm are lacking. In contrast, Lynce et al. reported a trend toward poorer survival in patients with early TNBC and gBRCAm compared to those without gBRCAm, suggesting that wider BRCA testing is important for the proper selection of adjuvant therapy [46,47]. Future studies are warranted to explore whether using both olaparib and capecitabine, in combination or in sequence, is necessary.

Most claims-derived HR+ patients (78%) received olaparib with other therapies, mainly hormonal therapy. Per the monarchE trial results, adjuvant abemaciclib with hormonal treatment was given to high-risk HR+ eBC patients, including a subset of those with gBRCAm. Although international guidelines recommend sequencing olaparib before CDK4/6 inhibitors [25,26], we did not observe any combinations of olaparib with abemaciclib in our study. This may be attributable to the characteristics of our cohort, which primarily consisted of patients with claims-based TNBC and presumed gBRCAm status. Because claims data do not reliably capture BRCA mutation status, olaparib use among patients with non-metastatic HER2-negative disease was used as a proxy for confirmed gBRCAm status. Additionally, the relatively short follow-up period may have contributed to these findings. Due to the evidence of all combinations seen, the efficacy and safety analyses of these patterns should be further investigated in future studies.

### Limitations

We recognize the limitations in our design that should be considered when interpreting the generalizability of our findings. Claims data do not capture tumor stage or genetic test data, preventing identification of eBC based on staging criteria or confirmed gBRCAm status. Therefore, we used a non-metastatic cohort as a proxy for eBC and relied on olaparib prescription among non-metastatic HER2-negative patients as a surrogate indicator of gBRCAm status. This approach was based on olaparib’s FDA-approved adjuvant indication for patients with gBRCAm-confirmed disease, for whom mutation testing is a prerequisite in clinical practice. Real-world olaparib utilization in a non-metastatic context is likely to reflect a population that closely approximates the intended gBRCAm eBC patient population, although the possibility of misclassification cannot be fully excluded. Classification of HR, TNBC, and HER2 statuses was not done using primary biomarker information due to its absence in claims data and instead relied on treatment history as a claims-derived proxy (e.g., presence of endocrine therapy for HR+ tumors, HER2-directed therapy for HER2-positive tumors, absence of endocrine therapy for TNBC, etc.). The short identification period of approximately 20 months restricted long-term examination of utilization outcomes. Our adherence analysis assumed patients consumed medications based on pharmacy claims. Disenrolled patients were censored, potentially affecting treatment discontinuation. While we present discontinuation rates, our data limited our ability to determine the reasons for discontinuation, such as recurrence, toxicity, or death.

## 5. Conclusions

These real-world data indicate that olaparib users had low rates of dose reductions and permanent discontinuation, and patients maintained a good adherence profile during the recommended treatment duration. Our adherence analysis suggests that patient support in the initial months may enhance continuity of olaparib treatment. We identified key combination patterns with olaparib, including hormonal therapy (for claims-derived HR+ patients) and immunotherapy (for claims-derived TNBC patients), and no substantial concomitant use with capecitabine or abemaciclib.

## Figures and Tables

**Table 1:** Demographic and clinical characteristics of patients with eBC who were treated with olaparib.

		Overall		Stratified by Race and Ethnicity								Stratified by Claims-Derived HR/TNBC			
Characteristic	Metric	Olaparib Users with eBC		White Non-Hispanic		Hispanic or Latino		African American		Hispanic or Latino and African American, Plus All Other Races and Ethnicities Combined ^a^		HR + Overall		TNBC Overall	
		n = 176		n = 70		n = 21		n = 16		n = 55		n = 68		n = 108	
Age (years)															
	Mean, SD	46.5	(11.5)	49.0	(12.8)	41.9	(10.6)	48.3	(13.7)	45.3	(12.0)	43.6	(11.2)	48.3	(11.4)
	Median (P25, P75)	44.0	(38.0, 54.0)	48.0	(40.0, 57.0)	38.0	(36.0, 44.0)	47.0	(38.5, 59.5)	41.0	(37.0, 54.0)	40.0	(35.0, 51.5)	48.0	(40.0, 56.0)
	Min, Max	24	81	26	81	24	68	28	77	24	77	28	75	24	81
Age group (years)															
18–34	n, %	27	15.3%	10	14.3%	5	23.8%	3	18.8%	10	18.2%	16	23.5%	11	10.2%
35–44	n, %	63	35.8%	18	25.7%	11	52.4%	5	31.3%	23	41.8%	29	42.6%	34	31.5%
45–54	n, %	43	24.4%	19	27.1%	2	9.5%	3	18.8%	9	16.4%	11	16.2%	32	29.6%
55–64	n, %	31	17.6%	15	21.4%	2	9.5%	3	18.8%	9	16.4%	8	11.8%	23	21.3%
65+	n, %	12	6.8%	8	11.4%	1	4.8%	2	12.5%	4	7.3%	4	5.9%	8	7.4%
Female	n, %	176	100.0%	70	100.0%	21	100.0%	16	100.0%	55	100.0%	68	100.0%	108	100.0%
Race and ethnicity															
White non-Hispanic	n, %	70	39.8%	-	-	-	-	-	-	-	-	25	36.8%	45	41.7%
African American	n, %	16	9.1%	-	-	-	-	-	-	-	-	5	7.4%	11	10.2%
Asian and Pacific Islander	n, %	4	2.3%	-	-	-	-	-	-	-	-	2	2.9%	2	1.9%
Hispanic or Latino	n, %	21	11.9%	-	-	-	-	-	-	-	-	9	13.2%	12	11.1%
Other	n, %	14	8.0%	-	-	-	-	-	-	-	-	7	10.3%	7	6.5%
Unknown/missing	n, %	51	29.0%	-	-	-	-	-	-	-	-	20	29.4%	31	28.7%
US Census geographic region															
Northeast	n, %	36	20.5%	17	24.3%	2	9.5%	4	25.0%	7	12.7%	15	22.1%	21	19.4%
West	n, %	32	18.2%	14	20.0%	5	23.8%	3	18.8%	14	25.5%	13	19.1%	19	17.6%
Midwest	n, %	37	21.0%	19	27.1%	4	19.0%	4	25.0%	11	20.0%	14	20.6%	23	21.3%
South	n, %	71	40.3%	20	28.6%	10	47.6%	5	31.3%	23	41.8%	26	38.2%	45	41.7%
Insurance payer type															
Commercial	n, %	117	66.5%	47	67.1%	8	38.1%	6	37.5%	26	47.3%	52	76.5%	65	60.2%
Medicare	n, %	18	10.2%	11	15.7%	1	4.8%	2	12.5%	5	9.1%	4	5.9%	14	13.0%
Medicaid	n, %	40	22.7%	12	17.1%	12	57.1%	8	50.0%	23	41.8%	12	17.6%	28	25.9%
Unknown/missing	n, %	1	0.6%	0	0.0%	0	0.0%	0	0.0%	1	1.8%	0	0.0%	1	0.9%
Cancer-adapted CCI ^b^ (continuous)															
	Mean, SD	0.8	(1.3)	0.8	(1.1)	1.5	(2.4)	0.5	(0.9)	1.0	(1.7)	0.7	(1.2)	0.9	(1.4)
	Median (P25, P75)	0.0	(0.0, 1.0)	0.0	(0.0, 1.0)	0.0	(0.0, 2.0)	0.0	(0.0, 1.0)	0.0	(0.0, 2.0)	0.0	(0.0, 1.0)	0.0	(0.0, 1.0)
	Min, Max	0	8	0	5	0	8	0	3	0	8	0	6	0	8
Cancer-adapted CCI (categorical)															
0	n, %	108	61.4%	40	57.1%	12	57.1%	11	68.8%	33	60.0%	43	63.2%	65	60.2%
1	n, %	27	15.3%	14	20.0%	2	9.5%	3	18.8%	7	12.7%	9	13.2%	18	16.7%
2	n, %	23	13.1%	11	15.7%	3	14.3%	1	6.3%	6	10.9%	10	14.7%	13	12.0%
3+	n, %	18	10.2%	5	7.1%	4	19.0%	1	6.3%	9	16.4%	6	8.8%	12	11.1%
Comorbidities															
Hypertension	n, %	66	37.5%	33	47.1%	7	33.3%	11	68.8%	22	40.0%	19	27.9%	47	43.5%
Depression	n, %	50	28.4%	23	32.9%	2	9.5%	6	37.5%	13	23.6%	20	29.4%	30	27.8%
Dyslipidemia	n, %	41	23.3%	20	28.6%	5	23.8%	5	31.3%	13	23.6%	12	17.6%	29	26.9%
Hypothyroidism	n, %	33	18.8%	16	22.9%	4	19.0%	2	12.5%	11	20.0%	14	20.6%	19	17.6%
Asthma	n, %	28	15.9%	12	17.1%	4	19.0%	3	18.8%	9	16.4%	9	13.2%	19	17.6%
Diabetes	n, %	21	11.9%	10	14.3%	3	14.3%	2	12.5%	8	14.5%	8	11.8%	13	12.0%
Osteoporosis	n, %	8	4.5%	7	10.0%	0	0.0%	0	0.0%	0	0.0%	1	1.5%	7	6.5%
Heart failure	n, %	4	2.3%	2	2.9%	1	4.8%	0	0.0%	1	1.8%	0	0.0%	4	3.7%
Ischemic heart disease	n, %	3	1.7%	3	4.3%	0	0.0%	0	0.0%	0	0.0%	0	0.0%	3	2.8%
Chronic obstructive pulmonary disease	n, %	3	1.7%	1	1.4%	0	0.0%	0	0.0%	1	1.8%	1	1.5%	2	1.9%
Treatment history prior to index date															
Type of BC surgery															
Lumpectomy only	n, %	27	15.3%	8	11.4%	3	14.3%	4	25.0%	10	18.2%	6	8.8%	21	19.4%
Mastectomy only	n, %	131	74.4%	54	77.1%	17	81.0%	10	62.5%	40	72.7%	50	73.5%	81	75.0%
Lumpectomy AND mastectomy	n, %	18	10.2%	8	11.4%	1	4.8%	2	12.5%	5	9.1%	12	17.6%	6	5.6%
Time to first surgery from first BC Dx date (days)	Mean, SD	160.1	(90.1)	152.7	(91.5)	179.3	(81.7)	163.6	(109.3)	176.0	(84.5)	135.3	(93.0)	175.6	(85.0)
	Median (P25, P75)	189.0	(57.5, 224.5)	189.0	(43.0, 220.0)	191.0	(127.0, 252.0)	210.0	(41.0, 239.5)	195.0	(127.0, 238.0)	173.0	(34.5, 209.0)	201.0	(126.0, 230.0)
	Min, Max	0	355	0	317	3	288	0	345	0	345	0	355	0	345
Time to lumpectomy from first BC Dx date (days) (among those who had lumpectomy only)	Mean, SD	130.0	(95.1)	113.9	(103.0)	95.3	(121.5)	173.0	(116.8)	152.3	(99.2)	87.3	(83.8)	142.2	(96.4)
	Median (P25, P75)	162.0	(36.0, 212.0)	76.0	(39.0, 195.0)	50.0	(3.0, 233.0)	222.0	(102.5, 243.5)	189.0	(50.0, 233.0)	55.0	(29.0, 173.0)	165.0	(42.0, 214.0)
	Min, Max	0	292	0	292	3	233	0	249	0	249	3	210	0	292
Time to mastectomy from first BC Dx date (days) (among those who had mastectomy only)	Mean, SD	177.7	(80.2)	174.7	(80.8)	197.2	(68.3)	188.2	(100.8)	194.3	(72.7)	154.0	(84.5)	192.4	(74.3)
	Median (P25, P75)	197.0	(155.0, 231.0)	199.0	(159.0, 223.0)	195.0	(161.0, 252.0)	222.0	(110.0, 240.0)	200.0	(162.5, 246.0)	187.0	(56.0, 220.0)	207.0	(181.0, 238.0)
	Min, Max	0	345	15	317	31	288	8	345	8	345	8	285	0	345
Time to first surgery from first BC Dx date (days) (among those who had both types)	Mean, SD	76.3	(96.3)	43.0	(56.0)	127.0	(.)	21.5	(2.1)	77.6	(77.7)	81.3	(108.1)	66.3	(75.1)
	Median (P25, P75)	34.0	(20.0, 127.0)	34.0	(10.0, 40.5)	127.0	(127.0, 127.0)	22.0	(20.0, 23.0)	27.0	(23.0, 127.0)	34.0	(23.5, 110.0)	29.0	(15.0, 127.0)
	Min, Max	0	355	0	176	127	127	20	23	20	191	0	355	7	191
Radiation, ^c^ any	n, %	38	21.6%	8	11.4%	4	19.0%	6	37.5%	13	23.6%	14	20.6%	24	22.2%
Neoadjuvant AND adjuvant	n, %	0	0.0%	0	0.0%	0	0.0%	0	0.0%	0	0.0%	0	0.0%	0	0.0%
Adjuvant ONLY	n, % ^c^	38	100.0%	8	100.0%	4	100.0%	6	100.0%	13	100.0%	14	100.0%	24	100.0%
Neoadjuvant ONLY	n, %	0	0.0%	0	0.0%	0	0.0%	0	0.0%	0	0.0%	0	0.0%	0	0.0%
Type of radiation (from index BC Dx date to index date)—among those with receipt of radiation															
Beam	n, %	38	100.0%	8	100.0%	4	100.0%	6	100.0%	13	100.0%	14	100.0%	24	100.0%
Brachytherapy	n, %	1	2.6%	0	0.0%	0	0.0%	0	0.0%	0	0.0%	0	0.0%	1	4.2%
Stereotactic radiotherapy	n, %	0	0.0%	0	0.0%	0	0.0%	0	0.0%	0	0.0%	0	0.0%	0	0.0%
Others	n, %	0	0.0%	0	0.0%	0	0.0%	0	0.0%	0	0.0%	0	0.0%	0	0.0%
Neoadjuvant treatment (not mutually exclusive)	n, %	123	69.9%	47	67.1%	17	81.0%	10	62.5%	43	78.2%	40	58.8%	83	76.9%
Chemotherapy	n, %	120	68.2%	46	65.7%	16	76.2%	10	62.5%	41	74.5%	37	54.4%	83	76.9%
Hormonal	n, %	19	10.8%	8	11.4%	2	9.5%	3	18.8%	9	16.4%	19	27.9%	0	0.0%
Targeted ^d^	n, %	1	0.6%	0	0.0%	1	4.8%	0	0.0%	1	1.8%	1	1.5%	0	0.0%
CDK4/6i	n, %	0	0.0%	0	0.0%	0	0.0%	0	0.0%	0	0.0%	0	0.0%	0	0.0%
Non-CDK4/6i	n, %	1	0.6%	0	0.0%	1	4.8%	0	0.0%	1	1.8%	1	1.5%	0	0.0%
Immunotherapy	n, %	63	35.8%	26	37.1%	8	38.1%	5	31.3%	20	36.4%	12	17.6%	51	47.2%
Neoadjuvant treatment (combinations)															
Chemotherapy alone	n, %	48	27.3%	16	22.9%	8	38.1%	2	12.5%	16	29.1%	16	23.5%	32	29.6%
Chemoimmunotherapy	n, %	55	31.3%	23	32.9%	6	28.6%	5	31.3%	17	30.9%	4	5.9%	51	47.2%
Chemotherapy plus hormonal	n, %	9	5.1%	4	5.7%	0	0.0%	3	18.8%	5	9.1%	9	13.2%	0	0.0%
Chemoimmunotherapy plus hormonal	n, %	8	4.6%	3	4.3%	2	9.5%	0	0.0%	3	5.5%	8	11.8%	0	0.0%
Hormonal alone	n, %	2	1.1%	1	1.4%	0	0.0%	0	0.0%	1	1.8%	2	2.9%	0	0.0%
Targeted alone	n, %	1	0.6%	0	0.0%	1	4.8%	0	0.0%	1	1.8%	1	1.5%	0	0.0%

Key: BC—breast cancer; CCI—Charlson Comorbidity Index; CDK4/6i—cyclin-dependent kinase 4/6 inhibitor; Dx—diagnosis; eBC—early breast cancer; HR—hormone receptor; HR+—hormone-receptor-positive; Min—minimum; Max—maximum; P25—25th percentile; P75—75th percentile; SD—standard deviation; TNBC—triple-negative BC. ^a^ “All other races and ethnicities combined” includes the sum of Asian and Pacific Islander and Other (note: “Unknown” is excluded from this group). ^b^ Cancer-adapted CCI was a score calculated after excluding cancer covariates. ^c^ Radiation timing and type percentages are reported out of patients who had any radiation. ^d^ One patient received targeted treatment (i.e., bevacizumab).

**Table 2:** Utilization patterns of olaparib patients, stratified by race and ethnicity, and HR/TNBC status.

		Olaparib Users with eBC		Stratified by Race and Ethnicity								Stratified by Claims-Derived HR/TNBC Status			
Characteristic	Metric			White Non-Hispanic		Hispanic or Latino		African American		Hispanic or Latino and African American, Plus All Other Races and Ethnicities Combined ^a^		HR+ (Overall)		TNBC (Overall)	
		n = 176		n = 70		n = 21		n = 16		n = 55		n = 68		n = 108	
Distinct olaparib claims	Mean, SD	7.2	(5.2)	-	-	-	-	-	-	-	-	-	-	-	-
	Median (P25, P75)	7.0	(3.0, 11.0)	-	-	-	-	-	-	-	-	-	-	-	-
	Min, Max	1	33	-	-	-	-	-	-	-	-	-	-	-	-
Utilization patterns															
Treatment interruption (within 12 months after index)	n, %	70	39.8%	27	38.6%	8	38.1%	6	37.5%	22	40.0%	31	45.6%	39	36.1%
Treatment interruption (overall)	n, %	73	41.5%	28	40.0%	8	38.1%	7	43.8%	23	41.8%	32	47.1%	41	38.0%
Time to first treatment interruption (KM calculated, in months)	Median (95% CI)	12.7	(8.9, ne)	ne	(9.7, ne)	ne	(2.3, ne)	5.5	(2.2, ne)	12.0	(4.6, ne)	9.7	(3.3, ne)	ne	(10.8, ne)
Treatment discontinuation (within 12 months after index) ^b^	n, %	74	42.0%	32	45.7%	9	42.9%	7	43.8%	23	41.8%	23	33.8%	51	47.2%
Treatment discontinuation (overall)	n, %	85	48.3%	36	51.4%	11	52.4%	8	50.0%	28	50.9%	30	44.1%	55	50.9%
Time to first treatment discontinuation (KM calculated in months)	Median (95% CI)	11.0	(9.2, 12.2)	11.3	(4.9, 12.9)	9.4	(2.9, ne)	9.2	(2.0, 12.0)	10.0	(5.3, 12.2)	12.0	(9.2, 12.9)	10.0	(6.0, 12.0)
Composite of interruption and/or discontinuation (within 12 months after index)	n, %	128	72.7%	55	78.6%	14	66.7%	10	62.5%	38	69.1%	48	70.6%	80	74.1%
Composite of interruption and/or discontinuation (overall)	n, %	133	75.6%	56	80.0%	15	71.4%	11	68.8%	41	74.5%	50	73.5%	83	76.9%
Time to first event (interruption/discontinuation, in months)	Mean, SD	3.0	(3.1)	2.6	(2.8)	3.2	(3.5)	3.0	(3.3)	3.0	(3.3)	2.9	(3.1)	3.1	(3.1)
	Median (P25, P75)	1.9	(1.0, 3.5)	1.8	(1.0, 3.3)	2.3	(1.0, 3.3)	2.1	(1.0, 3.6)	1.9	(1.0, 3.4)	1.5	(1.0, 3.5)	1.9	(1.0, 3.6)
	Min, Max	0.5	12.7	0.5	12.7	0.5	12.6	0.5	12.0	0.5	12.6	0.5	12.2	0.5	12.7
Time to first event (KM calculated, in months)	Median (95% CI)	2.9	(2.1, 3.4)	2.0	(1.4, 3.3)	2.8	(1.0, 10.0)	3.1	(1.0, 5.5)	2.7	(1.1, 4.6)	2.7	(1.6, 4.1)	2.9	(2.1, 3.6)
Treatment re-initiation (within 12 months after index)	n, %	23	27.1%	6	16.7%	4	36.4%	2	25.0%	10	35.7%	9	30.0%	14	25.5%
Treatment re-initiation (overall) ^b^	n, %	26	30.6%	6	16.7%	5	45.5%	3	37.5%	13	46.4%	11	36.7%	15	27.3%
Time to first treatment re-initiation (from discontinuation date, in months)	Mean, SD	2.9	(1.2)	2.7	(1.1)	3.3	(1.4)	2.1	(0.2)	3.1	(1.4)	2.8	(0.8)	3.0	(1.5)
	Median (P25, P75)	2.5	(2.0, 3.1)	2.4	(2.0, 2.6)	2.9	(2.0, 4.4)	2.1	(1.9, 2.4)	2.8	(2.0, 3.1)	2.6	(2.2, 3.1)	2.1	(2.0, 4.9)
	Min, Max	1.9	6.2	1.9	5.0	1.9	5.1	1.9	2.4	1.9	6.2	1.9	4.4	1.9	6.2
Dosing patterns ^c^	n, %	166	94.3%	66	94.3%	20	95.2%	15	93.8%	52	94.5%	65	95.6%	101	93.5%
Dose reduction, any	n, %	33	19.9%	13	19.7%	4	20.0%	4	26.7%	11	21.2%	15	23.1%	18	17.8%
Number of dose reductions	Mean, SD	1.2	(0.4)	1.1	(0.3)	1.5	(0.6)	1.3	(0.5)	1.3	(0.5)	1.1	(0.4)	1.2	(0.4)
	Median (P25, P75)	1	(1.0, 1.0)	1	(1.0, 1.0)	1.5	(1.0, 2.0)	1	(1.0, 1.5)	1	(1.0, 2.0)	1	(1.0, 1.0)	1	(1.0, 1.0)
	Min, Max	1	2	1	2	1	2	1	2	1	2	1	2	1	2
One dose reduction	n, %	27	81.8%	12	92.3%	2	50.0%	3	75.0%	8	72.7%	13	86.7%	14	77.8%
Two dose reductions	n, %	6	18.2%	1	7.7%	2	50.0%	1	25.0%	3	27.3%	2	13.3%	4	22.2%
Three or more dose reductions	n, %	0	0.0%	0	0.0%	0	0.0%	0	0.0%	0	0.0%	0	0.0%	0	0.0%
Time to first dose reduction (in months)	Mean, SD	3.5	(2.6)	4.1	(2.7)	1.1	(1.1)	1.7	(1.1)	1.7	(1.1)	3.2	(2.6)	3.7	(2.6)
	Median (P25, P75)	2.6	(1.4, 4.3)	3.2	(2.3, 6.2)	1.1	(0.1, 2.1)	1.3	(1.1, 2.4)	1.4	(1.0, 2.4)	2.3	(1.4, 3.6)	3.0	(1.3, 6.2)
	Min, Max	0.1	8.3	0.9	8.3	0.1	2.2	1.0	3.3	0.1	3.6	0.1	8.1	0.1	8.3

Key: CI—confidence interval; eBC—early breast cancer; HR—hormone receptor; HR+—hormone-receptor-positive; KM—Kaplan–Meier; Min—minimum; Max—maximum; P25—25th percentile; P75—75th percentile; ne—not estimable; SD—standard deviation; TNBC—triple-negative breast cancer. ^a^ “All other races and ethnicities combined” includes the sum of Asian and Pacific Islander and Other (note: “Unknown” is excluded from this group). ^b^ Note: Among patients who discontinued olaparib before 12 months (n = 74), 26 patients re-initiated the treatment again during the study period, resulting in 48 patients who permanently discontinued. ^c^ For the dosing analysis, we excluded patients with missing quantity information in the pharmacy data.

**Table 3:** Adherence trends of olaparib patients, stratified by evidence of any neoadjuvant treatment, prior platinum-based treatment use and HR/TNBC status.

		Stratified Based on Neoadjuvant Treatment				Stratified Based on Platinum-Based Treatment				Stratified Based on Claims-Derived HR/TNBC Status			
Characteristic	Metric	Without Prior Neoadjuvant Treatment		Prior Neoadjuvant Treatment		With Neoadjuvant or Adjuvant Platinum Use		No Platinum Use		HR+		TNBC	
		n = 53		n = 123		n = 80		n = 96		n = 68		n = 108	
Treatment adherence													
Adherence over fixed time frame													
PDC coverage for 30 days		51	96.2%	120	97.6%	77	96.3%	94	97.9%	67	98.5%	104	96.3%
PDC ≥ 80% (binary)	n, %	44	86.3%	109	90.8%	69	89.6%	84	89.4%	55	82.1%	98	94.2%
PDC (continuous measure)	Mean, SD	0.9	(0.1)	1	(0.1)	1	(0.1)	1	(0.1)	0.9	(0.2)	1	(0.1)
	Median (P25, P75)	1	(1.0, 1.0)	1	(1.0, 1.0)	1	(1.0, 1.0)	1	(1.0, 1.0)	1	(1.0, 1.0)	1	(1.0, 1.0)
	Min, Max	0.5	1	0.5	1	0.5	1	0.5	1	0.5	1	0.5	1.0
PDC coverage for 60 days		41	77.4%	101	82.1%	66	82.5%	76	79.2%	54	79.4%	88	81.5%
PDC ≥ 80% (binary)	n, %	25	61.0%	79	78.2%	50	75.8%	54	71.1%	35	64.8%	69	78.4%
PDC (continuous measure)	Mean, SD	0.8	(0.2)	0.9	(0.2)	0.8	(0.2)	0.8	(0.2)	0.8	(0.2)	0.9	(0.2)
	Median (P25, P75)	0.9	(0.6, 1.0)	1	(0.8, 1.0)	0.9	(0.8, 1.0)	0.9	(0.7, 1.0)	0.9	(0.7, 1.0)	1	(0.8, 1.0)
	Min, Max	0.5	1	0.3	1	0.3	1	0.5	1	0.5	1	0.3	1.0
PDC coverage for 180 days		27	50.9%	70	56.9%	48	60.0%	49	51.0%	38	55.9%	59	54.6%
PDC ≥ 80% (binary)	n, %	14	51.9%	48	68.6%	30	62.5%	32	65.3%	22	57.9%	40	67.8%
PDC (continuous measure)	Mean, SD	0.8	(0.2)	0.8	(0.2)	0.8	(0.2)	0.8	(0.2)	0.8	(0.2)	0.8	(0.2)
	Median (P25, P75)	0.8	(0.7, 0.9)	0.9	(0.7, 1.0)	0.8	(0.7, 1.0)	0.9	(0.8, 1.0)	0.8	(0.7, 0.9)	0.9	(0.7, 1.0)
	Min, Max	0.2	1	0.2	1	0.2	1	0.2	1	0.3	1.0	0.2	1.0
PDC coverage for 365 days		10	18.9%	21	17.1%	13	16.3%	18	18.8%	12	17.6%	19	17.6%
PDC ≥ 80% (binary)	n, %	7	70.0%	12	57.1%	8	61.5%	11	61.1%	5	41.7%	14	73.7%
PDC (continuous measure)	Mean, SD	0.8	(0.1)	0.8	(0.2)	0.8	(0.1)	0.8	(0.2)	0.8	(0.1)	0.8	(0.2)
	Median (P25, P75)	0.9	(0.8, 0.9)	0.8	(0.7, 0.9)	0.8	(0.7, 0.9)	0.8	(0.7, 0.9)	0.8	(0.7, 0.9)	0.9	(0.8, 0.9)
	Min, Max	0.6	1	0.4	1	0.5	1	0.4	1	0.5	1.0	0.4	1.0
Adherence over variable days of olaparib use													
PDC ≥ 30 days of olaparib use		51	96.2%	120	97.6%	77	96.3%	94	97.9%	67	98.5%	104	96.3%
PDC ≥ 80% (binary)	n, %	32	62.7%	91	75.8%	52	67.5%	71	75.5%	43	64.2%	80	76.9%
PDC (continuous measure)	Mean, SD	0.8	(0.2)	0.9	(0.2)	0.8	(0.2)	0.9	(0.2)	0.8	(0.2)	0.9	(0.2)
	Median (P25, P75)	0.9	(0.7, 1.0)	0.9	(0.8, 1.0)	0.9	(0.7, 1.0)	0.9	(0.8, 1.0)	0.9	(0.7, 1.0)	0.9	(0.8, 1.0)
	Min, Max	0.3	1	0.3	1	0.3	1	0.3	1	0.3	1.0	0.3	1
PDC ≥ 60 days of olaparib use		41	77.4%	101	82.1%	66	82.5%	76	79.2%	54	79.4%	88	81.5%
PDC ≥ 80% (binary)	n, %	23	56.1%	74	73.3%	43	65.2%	54	71.1%	33	61.1%	64	72.7%
PDC (continuous measure)	Mean, SD	0.8	(0.2)	0.8	(0.2)	0.8	(0.2)	0.8	(0.2)	0.8	(0.2)	0.8	(0.2)
	Median (P25, P75)	0.9	(0.7, 0.9)	0.9	(0.8, 1.0)	0.9	(0.7, 0.9)	0.9	(0.8, 1.0)	0.9	(0.7, 1.0)	0.9	(0.8, 1.0)
	Min, Max	0.3	1	0.3	1	0.3	1	0.3	1	0.3	1.0	0.3	1.0
PDC ≥ 180 days of olaparib use		27	50.9%	70	56.9%	48	60.0%	49	51.0%	38	55.9%	59	54.6%
PDC ≥ 80% (binary)	n, %	17	63.0%	50	71.4%	31	64.6%	36	73.5%	24	63.2%	43	72.9%
PDC (continuous measure)	Mean, SD	0.8	(0.2)	0.8	(0.2)	0.8	(0.2)	0.8	(0.2)	0.8	(0.2)	0.8	(0.2)
	Median (P25, P75)	0.9	(0.8, 0.9)	0.9	(0.8, 0.9)	0.9	(0.7, 0.9)	0.9	(0.8, 1.0)	0.9	(0.7, 0.9)	0.9	(0.8, 0.9)
	Min, Max	0.3	1	0.3	1	0.3	1	0.3	1	0.3	1.0	0.3	1.0
PDC ≥ 365 days of olaparib use		10	18.9%	21	17.1%	13	16.3%	18	18.8%	12	17.6%	19	17.6%
PDC ≥ 80% (binary)	n, %	7	70.0%	12	57.1%	8	61.5%	11	61.1%	5	41.7%	14	73.7%
PDC (continuous measure)	Mean, SD	0.8	(0.1)	0.8	(0.2)	0.8	(0.1)	0.8	(0.2)	0.8	(0.1)	0.8	(0.2)
	Median (P25, P75)	0.9	(0.8, 0.9)	0.9	(0.7, 0.9)	0.9	(0.7, 0.9)	0.9	(0.7, 0.9)	0.8	(0.7, 0.9)	0.9	(0.8, 0.9)
	Min, Max	0.6	0.9	0.4	1	0.6	0.9	0.4	1	0.5	1.0	0.4	1.0

Key: Min—minimum; Max—maximum; P25—25th percentile; P75—75th percentile; HR—hormone receptor; HR+—hormone-receptor-positive; PDC—proportion of days covered; SD—standard deviation; TNBC—triple-negative BC.

## Data Availability

Data presented in this study are available upon reasonable request from the corresponding author.

## Supplementary Materials

The following supporting information can be downloaded at: https://www.mdpi.com/article/10.3390/curroncol33100589/s1. Table S1: Clinical characteristics stratified by any neoadjuvant treatment and neoadjuvant or adjuvant platinum-based treatment. Table S2: Utilization patterns stratified by any neoadjuvant treatment and neoadjuvant or adjuvant platinum-based treatment.

## Abbreviations

Adj	Adjuvant treatment
Adj C + H	Adjuvant chemotherapy plus hormonal therapy
Adj C + Imm	Adjuvant chemotherapy plus immunotherapy
Adj Chem	Adjuvant chemotherapy
Adj Horm	Adjuvant hormonal treatment
Adj Imm	Adjuvant immunotherapy
Adj Other	Adjuvant other treatment
BC	Breast cancer
BRCAm	BRCA mutation
CCI	Charlson Comorbidity Index
CDK4/6	Cyclin-dependent kinase 4/6
CDK4/6i	Cyclin-dependent kinase 4/6 inhibitor
Chem	Chemotherapy
CI	Confidence interval
CPT	Current Procedural Terminology
DC	Discontinued treatment
Dx	Diagnosis
eBC	Early breast cancer
FDA	Food and Drug Administration
gBRCAm	Germline mutations in *BRCA1* and/or *BRCA2* genes
HER2	Human epidermal growth factor receptor 2
HIPAA	Health Insurance Portability & Accountability Act
HR	Hormone receptor
HR+	Hormone-receptor-positive
ICD-10-CM	International Classification of Diseases, Tenth Revision, Clinical Modification
ICD-10-PCS	International Classification of Diseases, Tenth Revision, Procedure Coding System
Imm	Immunotherapy
KM	Kaplan–Meier
Max	Maximum
Min	Minimum
NCCN	National Comprehensive Cancer Network
NDC	National Drug Code
ne	Not estimable
Neoadj	Neoadjuvant treatment
Olap Mono	Olaparib monotherapy
Olap + H	Olaparib plus hormonal treatment
Olap + Imm	Olaparib plus immunotherapy
Olap + other	Olaparib plus other treatment
PARP	Poly (adenosine diphosphate-ribose) polymerase
PDC	Proportion of days covered
P25	Twenty-fifth percentile
P75	Seventy-fifth percentile
RT	Radiation therapy
SAS	Statistical Analysis Software
SD	Standard deviation
SEER	Surveillance, Epidemiology and End Results
Sub C + Imm + RT	Subsequent chemotherapy plus immunotherapy plus radiation therapy
Sub Chem	Subsequent chemotherapy
Sub H + Imm	Subsequent hormonal plus immunotherapy
Sub Horm	Subsequent hormonal therapy
Sub Imm	Subsequent immunotherapy
Sub Imm + RT	Subsequent immunotherapy plus radiation therapy
Sub Targ	Subsequent targeted therapy
Subseq	Subsequent treatment
TNBC	Triple-negative breast cancer
Tx	Treatment
US	United States
