# Supplementary material for: Real-World Treatment Utilization and Patterns of Adjuvant Olaparib for Early-Stage Breast Cancer: Insights from the Komodo Research Database
Source: Curr Oncol. 2026 Sep 30;33(10):589. doi: 10.3390/curroncol33100589 (PMC13702983; doi:10.3390/curroncol33100589)
Supplement: Supplementary file 1 [file curroncol-33-00589-s001.zip › curroncol-4485453-supplementary.pdf]

SUPPLEMENTAL MATERIALS

Table S1. Clinical characteristics stratified by any neoadjuvant treatment and neoadjuvant or adjuvant platinum-based treatment

		Stratified by prior Tx[a]							
**Characteristic**	**Metric**	**Without prior neoadjuvant Tx**		**Prior neoadjuvant Tx**		**With neoadjuvant or adjuvant platinum use**		**No platinum use**	
		n=53		n=123		n=80		n=96	
**Cancer-adapted CCI[b]**									
	Mean, SD	0.8	(1.3)	0.8	(1.3)	0.7	(1.1)	0.9	(1.5)
	Median (P25, P75)	0.0	(0.0, 1.0)	0.0	(0.0, 1.0)	0.0	(0.0, 1.0)	0.0	(0.0, 1.0)
	Min, Max	0	8	0	6	0	5	0	8
**Cancer-adapted CCI**									
0	n, %	28	52.8%	80	65.0%	51	63.8%	57	59.4%
1	n, %	14	26.4%	13	10.6%	11	13.8%	16	16.7%
2	n, %	9	17.0%	14	11.4%	10	12.5%	13	13.5%
3+	n, %	2	3.8%	16	13.0%	8	10.0%	10	10.4%
**Comorbidities**									
Dyslipidemia	n, %	12	22.6%	29	23.6%	17	21.3%	24	25.0%
Hypertension	n, %	25	47.2%	41	33.3%	23	28.8%	43	44.8%
Ischemic heart disease	n, %	1	1.9%	2	1.6%	2	2.5%	1	1.0%
Heart failure	n, %	2	3.8%	2	1.6%	2	2.5%	2	2.1%
Diabetes	n, %	4	7.5%	17	13.8%	12	15.0%	9	9.4%
Osteoporosis	n, %	3	5.7%	5	4.1%	4	5.0%	4	4.2%
Hypothyroidism	n, %	10	18.9%	23	18.7%	18	22.5%	15	15.6%
Depression	n, %	18	34.0%	32	26.0%	26	32.5%	24	25.0%

Characteristic	Metric	Stratified by prior Tx[a]							
		Without prior neoadjuvant Tx		Prior neoadjuvant Tx		With neoadjuvant or adjuvant platinum use		No platinum use	
		n=53		n=123		n=80		n=96	
Asthma	n, %	8	15.1%	20	16.3%	14	17.5%	14	14.6%
COPD	n, %	0	0.0%	3	2.4%	1	1.3%	2	2.1%
**Tx history prior to index date**									
Type of BC surgery									
Lumpectomy only	n, %	10	18.9%	17	13.8%	7	8.8%	20	20.8%
Mastectomy only	n, %	31	58.5%	100	81.3%	67	83.8%	64	66.7%
Lumpectomy AND Mastectomy	n, %	12	22.6%	6	4.9%	6	7.5%	12	12.5%
Time to first surgery from first BC Dx date (days)	Mean, SD	46.8	(52.3)	208.9	(50.1)	203.1	(72.1)	124.2	(88.1)
	Median (P25, P75)	34.0	(20.0, 48.0)	210.0	(187.0, 238.0)	214.0	(189.0, 238.5)	151.0	(35.5, 196.0)
	Min, Max	0	285	13	355	3	355	0	288
Time to lumpectomy from first BC Dx date (days) (*among those who had lumpectomy only*)	Mean, SD	24.1	(21.7)	192.4	(57.3)	196.1	(93.1)	106.9	(86.4)
	Median (P25, P75)	26.0	(3.0, 42.0)	210.0	(165.0, 230.0)	230.0	(165.0, 238.0)	82.0	(32.5, 192.5)
	Min, Max	0	59	50	292	3	292	0	249
Time to mastectomy from first BC Dx date (days) (*among those who had mastectomy only*)	Mean, SD	62.4	(62.7)	213.5	(42.5)	210.6	(59.0)	143.3	(85.3)
	Median (P25, P75)	39.0	(29.0, 72.0)	211.0	(189.0, 239.5)	215.0	(191.0, 240.0)	164.0	(51.5, 202.5)
	Min, Max	0	285	55	345	8	345	0	288

		Stratified by prior Tx[a]							
Characteristic	Metric	Without prior neoadjuvant Tx		Prior neoadjuvant Tx		With neoadjuvant or adjuvant platinum use		No platinum use	
		n=53		n=123		n=80		n=96	
Time to first surgery from first BC Dx date (days) (*among those who had both types*)	Mean, SD	25.3	(13.0)	178.3	(111.6)	127.2	(134.4)	50.9	(63.3)
	Median (P25, P75)	28.0	(17.5, 35.0)	184.0	(127.0, 208.0)	81.0	(23.0, 208.0)	31.0	(16.5, 40.5)
	Min, Max	0	44	13	355	15	355	0	191
Radiation, any	n, %	13	24.5%	25	20.3%	18	22.5%	20	20.8%
Neoadjuvant AND adjuvant	n, %	0	0.0%	0	0.0%	0	0.0%	0	0.0%
Adjuvant ONLY	n, %[c]	13	100.0%	25	100.0%	18	100.0%	20	100.0%
Neoadjuvant ONLY	n, %	0	0.0%	0	0.0%	0	0.0%	0	0.0%
Type of radiation (*from index BC Dx date to index date*)									
Beam	n, %	13	100.0%	25	100.0%	18	100.0%	20	100.0%
Brachytherapy	n, %	0	0.0%	1	4.0%	0	0.0%	1	5.0%
Stereotactic radiosurgery	n, %	0	0.0%	0	0.0%	0	0.0%	0	0.0%
Others	n, %	0	0.0%	0	0.0%	0	0.0%	0	0.0%
Neoadjuvant Tx	n, %	-	-	123	100.0%	72	90.0%	51	53.1%
Chemotherapy	n, %	-	-	120	97.6%	72	90.0%	48	50.0%
Hormonal	n, %	-	-	19	15.4%	12	15.0%	7	7.3%
Targeted	n, %	-	-	1	0.8%	0	0.0%	1	1.0%
CDK4/6i therapy	n, %	-	-	0	0.0%	0	0.0%	0	0.0%
Non-CDK4/6i therapy	n, %	-	-	1	0.8%	0	0.0%	1	1.0%
Immunotherapy	n, %	-	-	63	51.2%	56	70.0%	7	7.3%

Key: BC – breast cancer; CCI – Charlson Comorbidity Index; CDK4/6i – cyclin-dependent kinase inhibitor; COPD – chronic obstructive pulmonary disease; Dx – diagnosis; Min – minimum; Max – maximum; P25 – 25th percentile; P75 – 75th percentile; SD – standard deviation; Tx – treatment.

[a] Prior Tx includes any treatment (chemotherapy, hormonal, targeted, immunotherapy) received prior to index date.

[b] Cancer-adapted CCI is a score calculated after excluding cancer covariates.

[c] Radiation timing and type percentages are reported out of patients who had any radiation.

Table S2. Utilization patterns stratified by any neoadjuvant treatment and neoadjuvant or adjuvant platinum-based treatment

		Stratified by neoadjuvant Tx				Stratified by neoadjuvant or adjuvant platinum-based Tx			
**Characteristic**	**Metric**	**Without prior neoadjuvant Tx**		**Prior neoadjuvant Tx**		**With neoadjuvant or adjuvant platinum use**		**No platinum use**	
		n=53		n=123		n=80		n=96	
**Utilization patterns**									
Treatment interruption (within 12 months after index)	n, %	23	43.4%	47	38.2%	34	42.5%	36	37.5%
Treatment interruption (overall)	n, %	23	43.4%	50	40.7%	36	45.0%	37	38.5%
Time to first treatment interruption	Mean, SD	64.3	(62.3)	110.7	(103.6)	107.2	(108.6)	85.2	(78.8)
	Median (P25, P75)	29	(29.0, 94.0)	70.5	(30.0, 147.0)	68	(29.0, 138.5)	61	(29.0, 102.0)
	Min, Max	14	296	14	386	14	386	14	366
Time to first treatment interruption (KM-calculated)	Median (95% CI)	ne	(94.0, ne)	386	(272.0, ne)	366	(180.0, ne)	ne	(251.0, ne)
Treatment discontinuation (within 12 months after index)[a]	n, %	21	39.6%	53	43.1%	38	47.5%	36	37.5%
Treatment discontinuation (overall)	n, %	27	50.9%	58	47.2%	41	51.3%	44	45.8%
Time to first treatment discontinuation	Mean, SD	177.1	(151.0)	132.8	(129.3)	125.4	(124.2)	166.9	(146.9)
	Median (P25, P75)	107	(29.0, 352.0)	65.5	(29.0, 236.0)	68	(29.0, 158.0)	100	(30.0, 320.5)
	Min, Max	14	437	14	484	14	484	14	437
Time to first treatment discontinuation (KM-calculated)	Median (95% CI)	342	(162.0, 379.0)	330	(253.0, 375.0)	281	(149.0, 381.0)	342	(285.0, 379.0)
Composite of interruption and/or discontinuation (within 12 months after index)	n, %	40	75.5%	88	71.5%	63	78.8%	65	67.7%
Composite of interruption and discontinuation (overall)	n, %	40	75.5%	93	75.6%	65	81.3%	68	70.8%

		Stratified by neoadjuvant Tx				Stratified by neoadjuvant or adjuvant platinum-based Tx			
**Characteristic**	**Metric**	**Without prior neoadjuvant Tx**		**Prior neoadjuvant Tx**		**With neoadjuvant or adjuvant platinum use**		**No platinum use**	
		n=53		n=123		n=80		n=96	
Time to first event (interruption/discontinuation)	Mean, SD	75.9	(78.5)	99	(99.8)	92.6	(96.0)	91.5	(93.2)
	Median (P25, P75)	37.5	(29.0, 101.0)	59	(29.0, 111.0)	59	(29.0, 110.0)	59	(29.0, 104.5)
	Min, Max	14	342	14	386	14	386	14	383
Time to first event (KM-calculated)	Median (95% CI)	68	(29.0, 107.0)	94	(68.0, 111.0)	70	(59.0, 105.0)	95	(61.0, 111.0)
Treatment re-initiation (within 12 months after index)	n, %	8	29.6%	15	25.9%	15	36.6%	8	18.2%
Treatment re-initiation (overall)[a]	n, %	8	29.6%	18	31.0%	15	36.6%	11	25.0%
Time to first treatment re-initiation (from discontinuation date)	Mean, SD	86.5	(33.6)	90.2	(39.7)	81.3	(35.5)	99.6	(38.6)
	Median (P25, P75)	73	(62.0, 106.5)	74.5	(61.0, 93.0)	62	(59.0, 91.0)	88	(72.0, 118.0)
	Min, Max	57	152	57	189	57	155	62	189
Time to first treatment re-initiation (KM-calculated)	Median (95% CI)	ne	(118.0, ne)	ne	(189.0, ne)	ne	(135.0, ne)	ne	(ne, ne)
**Dosing patterns**[b]	n, %	50	94.3%	116	94.3%	75	93.8%	91	94.8%
Index dose of olaparib									
150 mg/day	n, %	0	0	1	0.9	1	1.3	0	0
300 mg/day	n, %	3	6	8	6.9	6	8	5	5.5
350-400 mg/day	n, %	2	4	3	2.6	2	2.7	3	3.3
450-500 mg/day	n, %	1	2	2	1.7	1	1.3	2	2.2
600 mg/day	n, %	44	88	102	87.9	65	86.7	81	89

Characteristic	Metric	Stratified by neoadjuvant Tx: Without prior neoadjuvant Tx		Stratified by neoadjuvant Tx: Prior neoadjuvant Tx		Stratified by neoadjuvant or adjuvant platinum-based Tx: With neoadjuvant or adjuvant platinum use		Stratified by neoadjuvant or adjuvant platinum-based Tx: No platinum use	
		n=53		n=123		n=80		n=96	
Dose reduction, any	n, %	10	20.0%	23	19.8%	15	20.0%	18	19.8%
Number of dose reductions	Mean, SD	1.2	(0.4)	1.2	(0.4)	1.1	(0.4)	1.2	(0.4)
	Median (P25, P75)	1	(1.0, 1.0)	1	(1.0, 1.0)	1	(1.0, 1.0)	1	(1.0, 1.0)
	Min, Max	1	2	1	2	1	2	1	2
1 dose reduction	n, %	8	80.0%	19	82.6%	13	86.7%	14	77.8%
2 dose reductions	n, %	2	20.0%	4	17.4%	2	13.3%	4	22.2%
3 or more dose reductions	n, %	0	0.0%	0	0.0%	0	0.0%	0	0.0%
Time to first dose reduction	Mean, SD	84.9	(62.5)	113.7	(85.7)	114.9	(87.5)	96.7	(73.7)
	Median (P25, P75)	72	(44.0, 78.0)	93	(41.0, 196.0)	93	(43.0, 196.0)	76.5	(37.0, 110.0)
	Min, Max	30	244	4	251	4	251	4	244
Time to first dose reduction (KM-calculated)	Median (95% CI)	ne	(ne, ne)	ne	(ne, ne)	ne	(ne, ne)	ne	(ne, ne)

Key: CI – confidence interval; Min – minimum; Max – maximum; P25 – 25th percentile; P75 – 75th percentile; KM – Kaplan-Meier; ne – not estimable; SD – standard deviation; Tx – treatment.

[a] Note: Among patients who discontinued olaparib before 12 months (n=21), 8 patients did not re-initiate treatment again during the study period, resulting in 13 patients who permanently discontinued.

[b] Note: For the dosing analysis, we excluded patients due to missing quantity information in the pharmacy data.
